# Characteristics of nucleosomes and linker DNA regions on the genome of the basidiomycete *Mixia osmundae* revealed by mono- and dinucleosome mapping

**DOI:** 10.1098/rsob.120043

**Published:** 2012-04

**Authors:** Hiromi Nishida, Shinji Kondo, Takashi Matsumoto, Yutaka Suzuki, Hirofumi Yoshikawa, Todd D. Taylor, Junta Sugiyama

**Affiliations:** 1 Agricultural Bioinformatics Research Unit, Graduate School of Agricultural and Life Sciences, University of Tokyo, Tokyo 113-8657, Japan; 2 Quantitative Biology Center, RIKEN, 1-7-22 Suehiro, Tsurumi, Yokohama, Kanagawa 230-0045, Japan; 3 Genome Research Center, NODAI Research Institute, University of Tokyo, Kashiwa 277-8562, Japan; 4 Department of Medical Genome Sciences, Graduate School of Frontier Sciences, University of Tokyo, Kashiwa 277-8562, Japan; 5 Department of Bioscience, Tokyo University of Agriculture, Tokyo 156-8502, Japan; 6 TechnoSuruga Laboratory Co., Ltd, Chiba Branch Office and Laboratory, Chiba 274-0822, Japan

**Keywords:** basidiomycetes, evolution, fungi, *Mixia osmundae*, nucleosome, transcription

## Abstract

We present findings on the nucleosomal arrangement in the genome of the basidiomycete *Mixia osmundae*, focusing on nucleosomal linker DNA regions. We have assembled the genomic sequences of *M. osmundae*, annotated genes and transcription start sites (TSSs) on the genome, and created a detailed nucleosome map based on sequencing mono- and dinucleosomal DNA fragments. The nucleosomal DNA length distribution of *M. osmundae* is similar to that of the filamentous ascomycete *Aspergillus fumigatus*, but differs from that of ascomycetous yeasts, strongly suggesting that nucleosome positioning has evolved primarily through neutral drift in fungal species. We found clear association between dinucleotide frequencies and linker DNA regions mapped as the midpoints of dinucleosomes. We also describe a unique pattern found in the nucleosome-depleted region upstream of the TSS observed in the dinucleosome map and the precursor status of dinucleosomes prior to the digestion into mononucleosomes by comparing the mono- and dinucleosome maps. We demonstrate that observation of dinucleosomes as well as of mononucleosomes is valuable in investigating nucleosomal organization of the genome.

## 2. Introduction

The fungal genus *Mixia* (Protomycetaceae, Taphrinales, Ascomycetes) was introduced by Kramer [1] based on a single species, *Taphrina osmundae* [2], parasitic on fronds of the Japanese royal fern *Osmunda japonica* in Japan. Subsequently, the new family Mixiaceae (Protomycetales) was also proposed by Kramer [3] to accommodate the single genus *Mixia*. In those days, almost all mycologists believed that *Mixia* is a member of the ascomycetes, related to the species of the Protomycetales or Taphrinales. In 1995, it was reported that *Mixia osmundae* is not a member of Ascomycota but of Basidiomycota, based on molecular (18S rRNA sequence analysis) and morphological (light microscopic, standard electron microscopic and transmission electron microscopic observations on sporogenous cells) characters [4]. According to the classification of Assembling the Fungal Tree of Life (AFTOL), at present, *M. osmundae* has been accommodated in the Mixiales, Mixiomycetes, Pucciniomycotina and Basidiomycota, [5] and has no close relatives [6]. It takes a unique position in basidiomycete phylogeny as an enigmatic fungus. We previously reported an initial genome assembly of this species comprising 4408 contigs (a total of 3 019 501 nt) [7].

In the present study, we refined the genome assembly with additional sequencing, and annotated coding genes and their transcription start sites (TSSs), based, respectively, on RNA-seq [8] and the oligo-capping method [9] by using the Illumina GAIIx sequencer. For the annotated genome, we created a detailed nucleosome map by sequencing more than 60 million mono- and dinucleosomal DNA fragments, and investigated the nucleosomal organization around genes showing distinct levels of expression.

A nucleosome consists of an octamer of histones, around which genomic DNA is wrapped in 1.65 turns [10]. In recent years, studies have revealed a large number of patterns and rules for nucleosomes in relation to transcriptional activity. Recent findings include strong conservation of the nucleosome positions in gene promoter regions when compared with other genomic regions [11–14], and distinct conservation in nucleosome positioning between gene promoter and gene body regions [15], leading to proposals to use nucleosome positioning to categorize gene promoters [16,17]. Although *in vitro* studies revealed the influence of DNA sequences of core histones on nucleosome positioning, *in vivo* studies have demonstrated that ATP-dependent trans-acting factors play a major role in arranging nucleosomes in living cells [18–23]. All these patterns and rules observed in nucleosome positioning on active gene promoters suggest a functional link to transcription, and there is a clear correlation between nucleosome positioning and gene expression. It remains to be determined, however, whether changes in nucleosome positioning are a major driving force in the evolution of gene expression [16,24,25] or not [26,27].

Previous comparative studies of nucleosome positions were performed mainly using Saccharomycotina species (ascomycetous yeasts) [16,24–27]. Although some genome sequences of Basidiomycota species have been published [28–35], there is no nucleosome mapping data for the basidiomycetes. Comparison of nucleosome positioning between the ascomycetes and phylogenetically distant *M. osmundae* is expected to provide valuable information about the issue (i.e*.* whether nucleosome positioning is a major force in the evolution of gene expression). In this study, we identified mono- and dinucleosome positions on the genome of *M. osmundae* and investigated their features around the TSSs in relation to the level of transcriptional activity.

## 3. Results and discussion

### 3.1. Comparison of coding genes between *Mixia osmundae* and other fungal species

Assembly of the newly sequenced and previous datasets resulted in 283 contigs (a total of 13 393 708 nt). These 283 DNA sequences have been deposited to the DDBJ database under accession numbers BABT02000001–BABT02000283. On the newly assembled genome, 6726 protein-coding sequences (CDSs) were identified by using RNA-seq (see §5 and electronic supplementary material, table S1).

**Figure 1.**
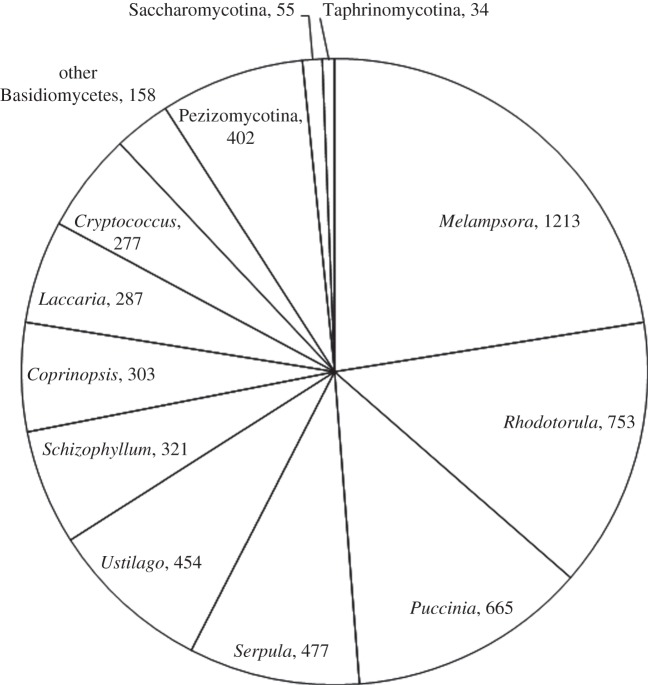
Pie chart of the genera with which each CDS of *Mixia osmundae* showed the highest similarity. The BLASTP program at NCBI (http://blast.ncbi.nlm.nih.gov/Blast.cgi) was used for the protein sequence similarity search. A total of 5399 CDSs (*E*-value < 10^−5^) were analysed.

The 6726 protein-coding sequences were aligned to the protein sequences of 80 fungal organisms using the BLASTP program in Fungal Genomes Central at NCBI (http://www.ncbi.nlm.nih.gov/projects/genome/guide/fungi/; electronic supplementary material, table S2). The BLASTP search revealed that 5399 (80.3%) of the 6726 CDSs had similar amino acid sequences with *E*-value < 10^−5^ among the fungal genes. Among the 5399 sequences, 4908 (90.9%), 402 (7.4%), 55 (1.0%) and 34 (0.6%) showed the highest similarity with proteins of Basidiomycota, Pezizomycotina (Ascomycota), Saccharomycotina (Ascomycota) and Taphrinomycotina (Ascomycota), respectively (figure 1). At the genus level, 1213 (22.5%), 753 (13.9%) and 665 (12.3%) showed the highest similarity with proteins of genera *Melampsora*, *Rhodotorula* and *Puccinia*, respectively (figure 1). As these three genera belong to Pucciniomycotina in Basidiomycota, *M. osmundae* is probably a member of Pucciniomycotina.

### 3.2. Comparison of size distribution of mono- and dinucleosomes of *Mixia osmundae* with *Aspergillus fumigatus* and *Saccharomyces cerevisiae*

**Figure 2.**
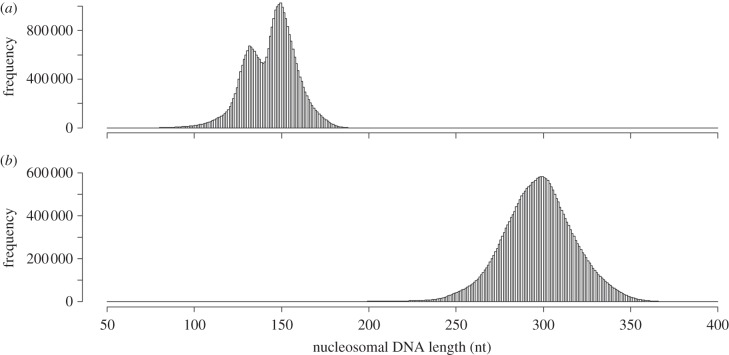
Histograms of (*a*) mononucleosomal and (*b*) dinucleosomal DNA fragment lengths of *Mixia osmundae*. The 30 386 916 mononucleosomal and 27 740 353 dinucleosomal DNA fragments were mapped to the genome. The distribution of the mononucleosomal DNA fragment lengths showed two peaks at 132 and 150 nt, whereas that of the dinucleosomal DNA fragment lengths showed only a single peak at 300 nt.

We determined the positions of the 30 381 113 mono- (range 80–230 nt) and 27 443 027 dinucleosomes (range 200–400 nt) on the genome of *M. osmundae* by mapping the mono- and dinucleosomal DNA fragments sequenced by the Illumina GAIIx. The nucleosome position data can be downloaded at http://www.iu.a.u-tokyo.ac.jp/~hnishida/data_Mixia.zip. The size distribution of the mononucleosomal DNA fragments had two peaks at 132 and 150 nt, whereas that of the dinucleosomal DNA fragments had only a single peak at 300 nt (figure 2).

As we reported previously, the double-peak distribution of mononucleosomal DNA fragments was observed in the filamentous ascomycete *Aspergillus fumigatus* [36]. The distribution of *A. fumigatus* mononucleosomal DNA fragments had two peaks at 135 and 150 nt [36]. In contrast, *Saccharomyces cerevisiae* had only a single peak at 163 nt [37]. In addition, the average mononucleosomal DNA length of 12 Saccharomycotina species is between 160 and 175 nt [16]. The nucleosomal DNA fragment length distribution depends on the micrococcal nuclease (MNase) treatment [38]. Based on the DNA ladder pattern on the agarose gel electrophoresis (electronic supplementary material, figure S1), we recognized that the digestion levels of MNase are similar between *M. osmundae* and *S. cerevisiae*. Thus, although *A. fumigatus* is phylogenetically closer to Saccharomycotina than *M. osmundae*, the distribution shape and size of mononucleosomal DNA fragments suggest that *M. osmundae* is closer to *A. fumigatus* than the ascomycetous yeasts (Saccharomycotina). This result adds support to the hypothesis that nucleosome positioning in fungal species has evolved primarily through neutral drift [26,27] and therefore fungi have species-specific nucleosome arrangements.

As we observed a loss of the longer peak at 150 nt in the highly transcribed (50 genes of highest level of expression) gene promoters of *A. fumigatus* [36], we examined whether the size of the mononucleosomes changes between the 50 genes of highest and lowest levels of expression in the upstream and downstream regions of the TSSs. However, in *M. osmundae*, the mononucleosomal DNA length distribution remained nearly invariant between distinct expression levels in both the upstream and downstream regions of the TSSs (electronic supplementary material, figure S2). The Mann–Whitney *U*-test yielded *p*-values of >0.7 and >0.11, respectively, for the upstream and downstream regions of the TSSs.

### 3.3. Comparison of dinucleosomes suggests a longer linker region in *Mixia osmundae* than in *Aspergillus fumigatus*

Prolonged treatment with MNase results in nearly complete cleavage of the total chromatin into mononucleosomes [39], suggesting that MNase-digested dinucleosomes are precursors of mononucleosomes [40]. Thus, dinucleosomal DNA fragments consist of two mononucleosomal DNAs and the linker DNA they flank. The distribution of dinucleosomal DNA lengths of *M. osmundae* had a single peak at 300 nt, whereas that of *A. fumigatus* had only a single peak at 285 nt [36]. As the mononucleosomal DNA lengths are nearly the same between the two fungi, the nucleosomal linker DNA lengths of *M. osmundae* appear to be approximately 15 nt longer than those of *A. fumigatus*.

### 3.4. Sizes of the mono- and dinucleosomes converge to their canonical sizes with increasing level of piles

**Figure 3.**
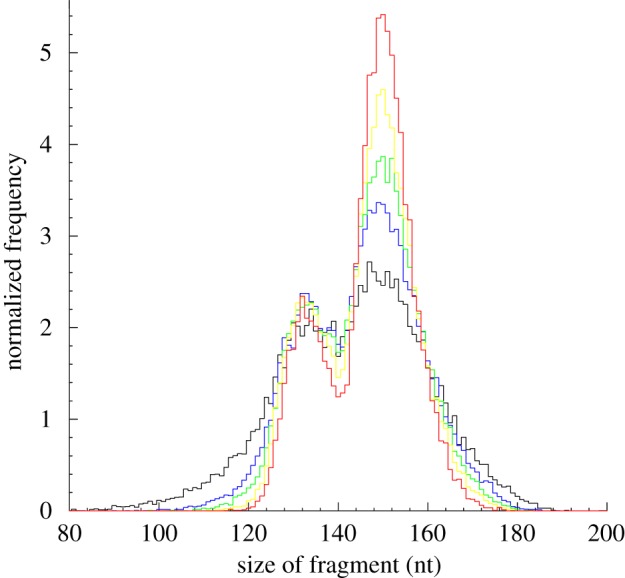
The sizes of both short and long mononucleosome groups converged to their respective mean sizes as the number of piles increased. Normalized distributions of mononucleosomal DNA fragments were compared between distinct pile levels. The same number (27 801) of mononucleosomal DNA fragments was used to compute the frequency distribution of each pile subset. The Mann–Whitney *U*-test yielded *p*-values of 0.005 or less in all the pairwise size comparisons. Red line, 10 piles; yellow line, 5 piles; green line, 3 piles; blue line, 2 piles; black line, 1 pile.

**Figure 4.**
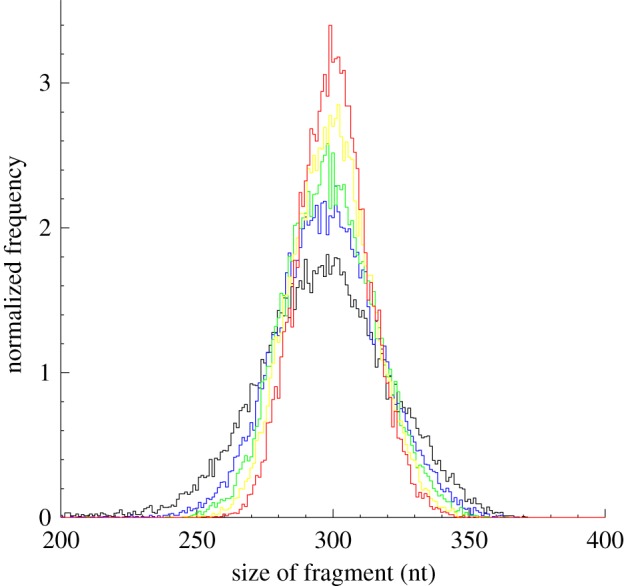
The sizes of dinucleosomes converged to the mean size with increasing pile level. Normalized distributions of dinucleosomal DNA fragments were compared between distinct pile levels. The same number (24 798) of dinucleosomal DNA fragments was used to compute the frequency distribution of each pile subset. The Mann–Whitney *U*-test yielded *p*-values of 0.0008 or less in all the pairwise size comparisons. Red line, 10 piles; yellow line, 5 piles; green line, 3 piles; blue line, 2 piles; black line, 1 pile.

To investigate potential differences in the nucleosome sizes between loosely and well-positioned nucleosomes, we examined how the double-peak distribution of mononucleosome lengths changes with increasing pile level (see §5). Interestingly, the sizes of both short- and long-mononucleosome groups converged to their respective mean sizes as the piles increased (figure 3). This convergence to the mean size was also observed in highly positioned dinucleosomes (figure 4). These sizes to which highly positioned mono- and dinucleosomes converge may be their canonical sizes taken to optimize functionality related to transcription, and the increase of the canonical portion may be required to optimize the nucleosome organization for the processes.

### 3.5. The dinucleosome position profile clarifies nucleosome depletion and suggests a longer linker DNA region in the upstream region of transcription start sites

**Figure 5.**
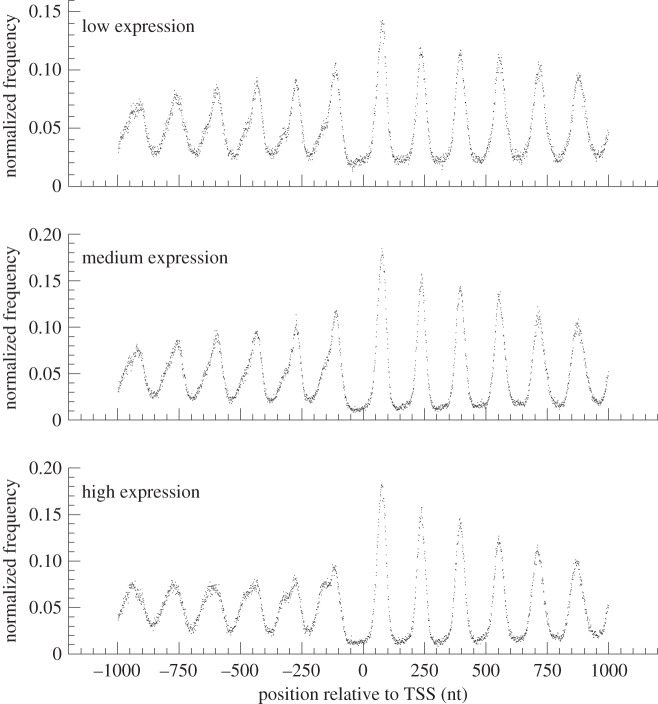
Profiles of the midpoints of highly positioned mononucleosomes of *Mixia osmundae* around the transcription start sites. The position profile of midpoints of highly positioned mononucleosomes in the vicinity of the TSS is compared between the three groups of genes showing distinct levels of expression. The nucleosomal midpoints of five or more piles were used.

**Figure 6.**
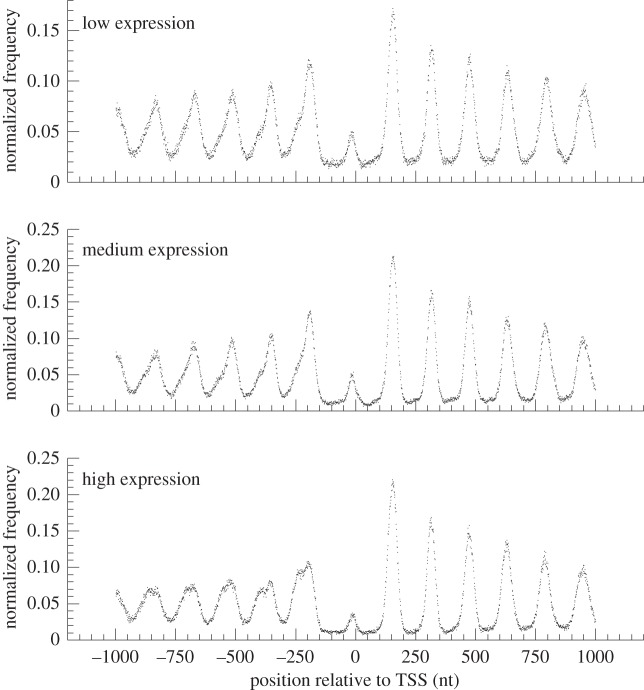
Profiles of the midpoints of highly positioned dinucleosomes of *Mixia osmundae* around the transcription start sites. The position profile of midpoints of highly positioned dinucleosomes in the vicinity of the TSS is compared between the three groups of genes showing distinct levels of expression. The nucleosomal midpoints of five or more piles were used.

The midpoint of a mononucleosome corresponds to the centre of the nucleosome core, whereas the midpoint of a dinucleosome lies in the linker DNA region flanked by two nucleosomes. Therefore, the peaks and bottoms of the midpoint profile of the mononucleosomes (figure 5) coincide, respectively, with the bottoms and peaks of the midpoint profile of dinucleosomes (figure 6). On the other hand, the interval between neighbouring peaks in the midpoint profile of dinucleosomes (figure 6) was identical to that of mononucleosomes (figure 5), indicating that the dinucleosomal DNA fragments were generated at random by MNase digestion. This result supports that MNase-digested dinucleosomes are precursors of mononucleosomes [40].

The nucleosome organization of *M. osmundae* around the TSSs (figure 5) was nearly identical to that of *S. cerevisiae* [18,23]. The highest peak of mononucleosome midpoints is found at position +1 on the downstream side of the TSS. Peak height decreases as the position moves away from the TSS on both the up- and downstream sides. The nucleosome position profile was sharper in the downstream region than in the upstream region of the TSS. Interestingly, in the upstream region of the TSS, genes with low expression levels showed a more clear phasing pattern of nucleosomes than genes with high levels of expression (figure 5; see §5 for the three sets of genes showing distinct levels of expression).

Nucleosome depletion upstream of the TSS was observed as a loss of dinucleosomes mapped at the positions −1 and +1. Note that the degree of loss of dinucleosomes mapped at the −1 and +1 positions increased with increasing level of gene expression. A *χ*^2^ test of frequencies of dinucleosomes mapped around position −1 (−200 to −1 nt from the TSS) yielded *p*-values of approximately 0 and <10^−15^, respectively, between the gene sets of low and medium expression levels, and between the sets of medium and high expression levels. The loss of dinucleosomes mapped to the −1 and +1 positions strongly suggests that MNase digests chromatins near nucleosome-depleted regions more quickly than other regions. It was supported by the fact that the interval size between the peaks of the −1 and +1 positions in the nucleosome midpoint profile (figure 5) was longer than the others.

### 3.6. Mono- and dinucleosome organization around the translational termination sites

Since a previous study reported a phasing pattern of nucleosomes around the transcription termination sites for a subset of yeast genes [41], we examined the mono- and dinucleosome organization around the 3′ ends of ORFs of the three gene sets of distinct expression levels. Some phasing was observed in regions internal to genes in both mono- and dinuceosome maps for the gene group of high expression (electronic supplementary material, figures S3 and S4). In addition, we found a prominent phasing of the nucleosome penultimate to the 3′ end of ORF, as observed earlier [41]. Surprisingly, the dinucleomes were clearly depleted in regions immediately downstream of the 3′ ends of ORFs. This notable depletion of dinucleosomes near the translational termination sites supports the regulation mechanism of terminal nuclesomes in transcriptional termination [42], and may be related to disassembling of the terminating RNA polymerase, assembling of anti-sense pre-initiation complexes and/or a gene looping mediated by transcription factor IIB (TFIIB) [41].

### 3.7. Enriched and depleted dinucleotide sequences in and around *Mixia osmundae* mono- and dinucleosomes

**Figure 7.**
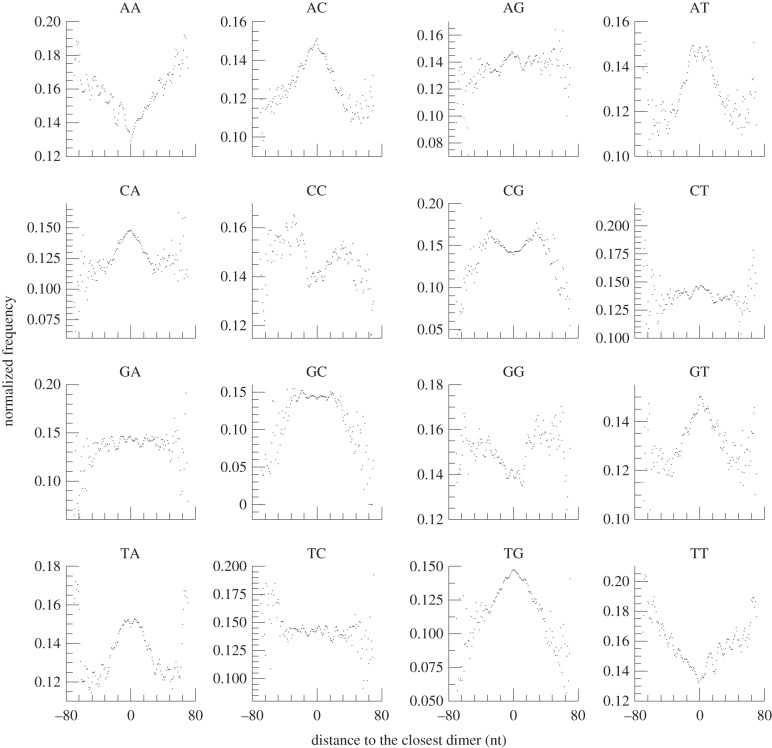
Enriched and depleted dinucleotides around the midpoints of highly positioned mononucleosomes of *Mixia osmundae*. Composite profiles of the distances between midpoints of highly positioned (five or more piles) mononucleosomes and their closest dinucleotides are shown. The *x*- and *y*-axes represent, respectively, the position relative to the TSS and the normalized frequencies of the distances from the nucleosomal midpoints to the closest dinucleotides. The frequencies of the distances were normalized to the average frequencies at all the genome positions and each of the 16 profiles was normalized to 100.

**Figure 8.**
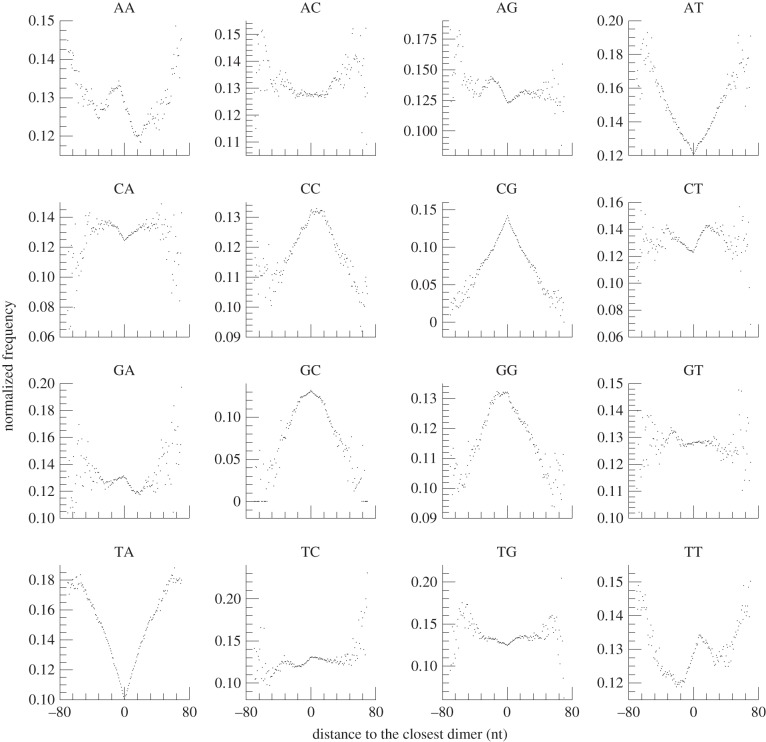
Enriched and depleted dinucleotides around the midpoints of highly positioned dinucleosomes of *Mixia osmundae*. Composite profiles of the distances between midpoints of highly positioned (five or more piles) dinucleosomes and their closest dinucleotides are shown. The *x*- and *y*-axes represent, respectively, the position relative to the TSS and the normalized frequencies of the distances from the midpoints of dinucleosomes to the closest dinucleotides. The frequencies of the distances were normalized to the average frequencies at all the genome positions and each of the 16 profiles was normalized to 100.

Previous studies reported that sequences rich in guanine (G) and cytosine (C) favour core nucleosomes, while sequences rich in adenine (A) and thymine (T) disfavour them, although some variations of preferred sequences exist between species [25]. We examined the biases of all 16 dinucleotide sequences in and around the midpoints of the mono- and dinucleosome positions by comparing the profiles of distances to the closest dinucleotides (see electronic supplementary material, figure S5, for definition of the closest dinucleotide). The enriched and depleted dinucleotides in and around the midpoints of the mononucleosome of *M. osmundae* showed features common to those of humans [22] (i.e. G/C-rich and A/T-rich sequences tend to be enriched, respectively, at the centres and edges of the mononucleosomes). We also examined dinucleotide enrichment in and around the dinucleosomes. Interestingly, there were clear preferences of dinucleotides associated with dinucleosomes, and the dinucleotides enriched or depleted at the midpoints of dinucleosomes differed from those found in and around the midpoints of mononucleosomes (figures 7 and 8). We believe this is the first report of DNA sequence preference of the nucleosomal linker DNA regions, inferred from the direct DNA sequence data. The dinucleotide sequence distributions are very different between the nucleosome-bound region and the nucleosomal linker region (figures 7 and 8). For example, the dinucleotide sequences CC, CG, GC and GG are enriched in the nucleosomal linker regions (figure 8), while AT and TA are depleted in the nucleosomal linker regions (figure 8). These dinucleotide sequences, AT and TA, are enriched in the nucleosome core regions (figure 7), while they become enriched in regions away from the midpoints of dinucleosomes towards their edges (figure 8; see electronic supplementary material, figure S6, for the profile in extended regions from the midpoints).

Since previous studies [43,44] used dinucleotide frequencies around the nucleosome midpoints to identify the dinucleotides favoured or disfavoured by the nucleosome cores, we also examined the dinucleotide frequencies around the highly positioned nucleosome midpoints (as shown in electronic supplementary material, figures S7 and S8). Some dinucleotides showed profiles distinct from that of distances to the closest dinucleotides. For example, enrichment of CC, CG, GC and GG found in the profile of distances to the closest dinucleotides was not observed in that of dinucleotide frequencies around midpoints of mononucleosomes, and depletion of AA and TT in the profile of distances to the closest dinucleotides is not observed in that of dinucleotide frequencies around the midpoints of dinucleosomes. This difference occurred since only a single dinucleotide closest to the midpoint of a nucleosome was considered in the profile of distances to the closest dinucleotides, whereas all the dinucleotides in regions flanking the midpoint were counted in computing dinucleotide frequencies. Thus, this difference between the two profiles indicates which of the closest dinucleotide to a position or the overall distribution around the position is a major determinant in the positioning of nucleosomes. Since many previous studies reported the enrichment of C/G-rich dinucleotides and depletion of A/T-rich dinucleotides in the nucleosome cores, this observation suggests that the closest dinucleotide is more important than its overall distribution in nucleosome positioning.

## 4. Conclusions

By effectively using the high-throughput datasets of mono- and dinucleosomal DNA fragments, we have identified characteristics of nucleosomal arrangement and linker DNA regions in the genome of the basidiomycete *M. osmundae,* which holds a unique phylogenic position in the evolution of fungal species. The double-peak distribution of mononucleosomal DNA lengths of *M. osmundae* resembles that of *A. fumigatus* and clearly differs from the single-peak distribution of ascomycetous yeasts. This strongly suggests that nucleosome positioning has evolved primarily through neutral drift in fungal species. Thus, the divergence of nucleosome positioning is not correlated with that of gene expression.

The profile of dinucleosomal midpoints around the TSS presents a unique view of the nucleosome-depleted region, which was observed as a loss of the first peak on the upstream side of the TSS. The degree of loss of the peak clearly correlates with gene expression levels. The loss of dinucleosomes mapped in the position upstream of the TSS implicates an extended size of linker DNAs in this region, and thus quicker digestion of the longer linkers when compared with those of other regions. Comparison of the phasing patterns of mono- and dinucleosomes around the TSS revealed random cleaving of dinucleosomal DNA fragments. This gives additional support to our previous observation that dinucleosomes are precursors of mononucleosomes prior to MNase digestion.

Importantly, we found clear sequence biases in linker DNA regions mapped by the midpoints of dinucleosomes. The propensity of the linkers to digestion is likely to be regulated at least in part by the arrangement of DNA sequences. This association of DNA sequences with uncleaved linkers, in addition to the unique arrangement of dinucleosomes near the TSS, may implicate some specific regulatory mechanism for the midpoint linkers of dinucleosomes.

We have demonstrated the great utility of dinucleosomes in investigating nucleosomal organization of the *M. osmundae* genome. Extension of this approach to other species and comparative studies will reveal important aspects of evolution of nucleosome organization.

## 5. Material and methods

### 5.1. *Mixia osmundae* culturing

*Mixia osmundae* IAM 14324 (i.e. JCM 22182, CBS 9802) was used in this study (for strain data, see [6]). The strain was cultivated in YM broth (yeast extract, 3 g; malt extract, 3 g; peptone, 5g; dextrose, 10 g; water, 1 l) at 25°C to an absorbance at 600 nm of 0.7 ml^−1^.

### 5.2. DNA sequencing

Genome sequencing was performed using the massively parallel Roche GS FLX Titanium DNA sequencer. Assembly of the reads was performed using the Newbler program (v. 2.3). The 5′-end sequences were determined using the oligo-capping method [9]. The cDNAs from total mRNA were sequenced using the Illumina Genome Analyzer IIx.

### 5.3. Nucleosome mapping

Cells were collected from 50 ml culture and resuspended in 250 µl of NP buffer (1 M sorbitol, 50 mM NaCl, 10 mM Tris–HCl at pH 7.4, 5 mM MgCl_2_, 1 mM CaCl_2_ and 0.075% Nonidet P40, with freshly added 1 mM β-mercaptoethanol and 500 µM spermidine). Cell suspensions were homogenized using a Covaris S2 manufacture. The homogenate was filled up to 2 ml with NP buffer and was divided into six aliquots of 300 μl each, and then MNase was added at concentrations of 0, 0.1, 0.5, 1, 3 and 5 U per sample. The digestion reactions were incubated at 37°C for 30 min, and were stopped by adding sodium dodecylsulphate to a final concentration of 1 per cent and ethylene diaminetetraacetic acid to a final concentration of 10 mM. Five microlitres of proteinase K solution (20 mg ml^−1^) was added and then the mixtures were incubated at 56°C for 1 h. Samples were phenol/chloroform-extracted, ethanol-precipitated and treated with RNase. To isolate the nucleosomal DNA fragments, electrophoresis was carried out on a 2 per cent agarose gel (electronic supplementary material, figure S1). The mononucleosomal and dinucleosomal DNA bands were excised and purified using the QIAquick Gel Extraction Kit (QIAGEN). Both ends (76 bases) of the DNA fragments treated with 5 U of MNase were sequenced using the Illumina GAIIx. The short read pairs were aligned to the genomic contigs of *M. osmundae* using SOAP2 [45]. Only uniquely mapped read pairs were used for the analysis.

### 5.4. Highly positioned mono- and dinucleosomes

Valouev *et al*. identified characteristics of mononucleosomes in the human genome, effectively using highly positioned nucleosomes, which were redundantly mapped (or piled) by high-throughput sequencing data of MNase-processed human genomic DNA [22]. They reported a profile of consistently positioned nucleosomes, correlation of phasograms (histograms of sizes of the nucleosome core + linker) with expression level, and sequence preferences associated with nucleosome cores. Following their approach, we investigated nucleosome positions redundantly mapped by the mono- and dinucleosome DNA fragments. Unless otherwise specified, we used subsets of the midpoints of mono- and dinucleosomes and positions of fragmental DNAs mapped by five or more sequence pairs (5-pile subsets) in the analyses (1 916 208 and 1 714 131 midpoints of mono- and dinucleosomal DNA fragments, respectively).

### 5.5. RNA-seq-based gene prediction

Using AUGUSTUS [46], a gene-prediction program based on alignment of expressed sequences to the genome, we determined the coding regions (CDSs) of the genes expressed on the genome of *M. osmundae*. We obtained 42 706 936 single reads (76 nt) by sequencing a total RNA sample of *M. osmundae* using Illumina GAIIx. Single reads of 34 962 178 could be aligned to the genome allowing five or less mismatches using BLAT [47]. Based on the coordinates mapped by the expressed sequences, AUGUSTUS predicted a total of 6569 genes (6462 complete and 107 partial genes). Since 148 genes showed alternative splicing forms, a total of 6726 transcripts were detected among the 6569 gene loci. We used the gene model trained for *Ustilago*, the closest basidiomycete to *M. osmundae* among those available in AUGUSTUS. A total of 13 546 fragments (1 kb each) of the *M. osmundae* genome contigs were aligned to the genome of *Ustilago maydis* 521 by BLAST [48]. About one-third (4157) of the 1 kb fragments of *M. osmundae* could be aligned to the *Ustilagao* genome at 68 ± 6% over 346 ± 230 nt.

### 5.6. Determination of levels of gene expression based on RNA-seq

The expression levels of the 6462 complete genes were determined based on the alignment of the RNA-seq reads to the genome. The expression level, in RPKM (reads mapped per kilobase of mature transcript per million of reads) [49], was computed counting the number of the reads mapped to the exons of each gene. According to the levels of expression thus determined, we made three gene groups of low (rank: 1–1000; 0.2 ≦ RPKM ≦ 14.6), medium (rank: 2732–3731; 41.3 ≦ RPKM ≦ 63.9) and high (rank: 5463–6462; 199 ≦ RPKM ≦ 22 400) level of expression of an equal size (1000 genes each).

### 5.7. Mapping of transcriptional start sites by the oligo-capping method

We determined the TSS of the expressed genes on the genome of *M. osmundae* by mapping the 5′ ends of full-length cDNAs obtained by the oligo-capping method [9]. We generated 17 750 643 single reads (36 bp each) of the 5′ ends by Illumina GAIIx. In total, 10 642 239 of them could be aligned to the genome by SOAP2 [45]. Typical profiles of the TSSs mapped near the translation start sites (TRSs) determined by AUGUSTUS are shown in electronic supplementary material, figure S9. We searched for the position mapped by the largest number of short reads within the 1 kb upstream region of the TRS of each gene, and the upstream position most intensely mapped by the 5′-end reads was used as the TSS of the gene. The number of oligo-capped reads mapped within the 1 kb upstream region of the TRS highly correlated with the RPKM values determined by RNA-seq. The 1 kb upstream regions of the three gene groups of low, medium and high level of expression were mapped, respectively, by 170,440 and 3500 oligo-capped reads, on average.

## Supplementary Material

Table S1 Summary of the numbers of reads generated by Illumina IIGx and mapped to the genome, nucleosome midpoints mapped by five or more read pairs and protein-coding genes determined

## Supplementary Material

Table S2 Fungal species names used in the comparative study

## Supplementary Material

Figures S1, S2, S3, S4, S5, S6, S7, S8, S9
