# Correction to ‘Characteristics of nucleosomes and linker DNA regions on the genome of the basidiomycete *Mixia osmundae* revealed by mono- and dinucleosome mapping’

**DOI:** 10.1098/rsob.120171

**Published:** 2012-12

**Authors:** Hiromi Nishida, Shinji Kondo, Takashi Matsumoto, Yutaka Suzuki, Hirofumi Yoshikawa, Todd D. Taylor, Junta Sugiyama

*Open Biol*. **2**. (Published online 4 April 2012). (doi:10.1098/rsob.120043)

The authors wish to correct their affiliation as below:

*New address* (third author listed): Genome Research Center, NODAI Research Institute, Tokyo University of Agriculture, Tokyo 156-8502, Japan.

*Incorrect*: Genome Research Center, NODAI Research Institute, University of Tokyo, Kashiwa 277-8562, Japan.

