# Supplementary material for: Characteristics of nucleosomes and linker DNA regions on the genome of the basidiomycete Mixia osmundae revealed by mono- and dinucleosome mapping
Source: Open Biol. 2012 Apr;2(4):120043. doi: 10.1098/rsob.120043 (PMC3376729; doi:10.1098/rsob.120043)
Supplement: Table S1 Summary of the numbers of reads generated by Illumina IIGx and mapped to the genome, nucleosome midpoints mapped by five or more read pairs and protein-coding genes determined [file rsob120043-s1.doc]

Supplementary Table S1-1. Numbers of reads generated by Illumina IIGx and mapped to the genome.

| Dataset | Mapped | Generated |
| --- | --- | --- |
| Mononucleosomal fragments (paired reads, 76 bp) | 30,381,113  (80 bp ≤ range ≤ 230 bp)  (with ≤ 2 mismatches/read) | 46,385,546 |
| Dinucleosomal fragments (paired reads, 76 bp) | 27,443,027  (200 bp ≤ range ≤ 400 bp)  (with ≤ 2 mismatches/read) | 30,821,169 |
| RNA-seq (single reads, 76 bp) | 34,962,178  (with ≤ 5 mismatches ) | 42,706,936 |
| Oligo-capped TSS (single reads, 36 bp) | 10,642,239  (with ≤ 2 mismatches) | 17,750,643 |

Supplementary Table S1-2. Numbers of nucleosome midpoints mapped by five or more read pairs.

| Dataset | Number |
| --- | --- |
| Mononucleosome midpoint | 1,916,208 |
| Dinucleosome midpoint | 1,714,131 |

Supplementary Table S1-3. Number of protein-coding genes determined by AUGUSTUS based on the RNA-seq.

| Gene class | Number of genes determined |
| --- | --- |
| Complete genes | 6,462 |
| Partial genes | 107 |
| Total number of gene loci | 6,569 |
| Genes subject to alternative loci | 148 |
| Total number of transcripts including splicing variants | 6,726 |
| Aligned to genes of 80 fungal species with *E*-value < 10^-5^ | 5,399 |
