# Supplementary material for: Characteristics of nucleosomes and linker DNA regions on the genome of the basidiomycete Mixia osmundae revealed by mono- and dinucleosome mapping
Source: Open Biol. 2012 Apr;2(4):120043. doi: 10.1098/rsob.120043 (PMC3376729; doi:10.1098/rsob.120043)
Supplement: Table S2 Fungal species names used in the comparative study [file rsob120043-s2.doc]

Supplementary Table S2. Fungal species names used in the comparative study.

43 species from Pezizomycotina, Ascomycota:

*Ajellomyces capsulatus*

*Ajellomyces dermatitidis*

*Arthroderma benhamiae*

*Arthroderma gypseum*

*Arthroderma otae*

*Aspergillus clavatus*

*Aspergillus flavus*

*Aspergillus fumigatus*

*Aspergillus nidulans*

*Aspergillus niger*

*Aspergillus terreus*

*Botryotinia fuckeliana*

*Chaetomium globosum*

*Chaetomium thermophilum*

*Coccidioides immitis*

*Coccidioides posadasii*

*Fusarium oxysporum*

*Gibberella zeae*

*Glomerella graminicola*

*Magnaporthe oryzae*

*Metarhizium acridum*

*Metarhizium anisopliae*

*Mycosphaerella graminicola*

*Nectria haematococca*

*Neosartorya fischeri*

*Neurospora crassa*

*Neurospora tetrasperma*

*Paracoccidioides brasiliensis*

*Penicillium chrysogenum*

*Penicillium marneffei*

*Phaeosphaeria nodorum*

*Podospora anserina*

*Pyrenophora teres*

*Pyrenophora tritici-repentis*

*Sclerotinia sclerotiorum*

*Talaromyces stipitatus*

*Trichoderma reesei*

*Trichophyton equinum*

*Trichophyton rubrum*

*Trichophyton tonsurans*

*Trichophyton verrucosum*

*Uncinocarpus reesii*

*Verticillium albo-atrum*

18 from Saccharomycotina, Ascomycota:

*Ashbya gossypii*

*Candida albicans*

*Candida dubliniensis*

*Candida glabrata*

*Candida tropicalis*

*Clavispora lusitaniae*

*Debaryomyces hansenii*

*Kluyveromyces lactis*

*Lachancea thermotolerans*

*Lodderomyces elongisporus*

*Meyerozyma guilliermondii*

*Pichia angusta*

*Pichia pastoris*

*Saccharomyces cerevisiae*

*Scheffersomyces stipitis*

*Vanderwaltozyma polyspora*

*Yarrowia lipolytica*

*Zygosaccharomyces rouxii*

2 from Taphrinomycotina, Ascomycota:

*Schizosaccharomyces japonicus*

*Schizosaccharomyces pombe*

13 from Basidiomycota:

*Coprinopsis cinerea*

*Cryptococcus gattii*

*Cryptococcus neoformans*

*Laccaria bicolor*

*Malassezia globosa*

*Melampsora larici-populina*

*Moniliophthora perniciosa*

*Postia placenta*

*Puccinia graminis*

*Rhodotorula glutinis*

*Schizophyllum commune*

*Serpula lacrymans*

*Ustilago maydis*

4 from Microsporidia:

*Encephalitozoon cuniculi*

*Encephalitozoon intestinalis*

*Enterocytozoon bieneusi*

*Nosema ceranae*
