# Supplementary material for: Characteristics of nucleosomes and linker DNA regions on the genome of the basidiomycete Mixia osmundae revealed by mono- and dinucleosome mapping
Source: Open Biol. 2012 Apr;2(4):120043. doi: 10.1098/rsob.120043 (PMC3376729; doi:10.1098/rsob.120043)

Supplementary Figure S1. Agarose gel electrophoresis of DNA fragments digested by different concentrations of micrococcal nuclease.

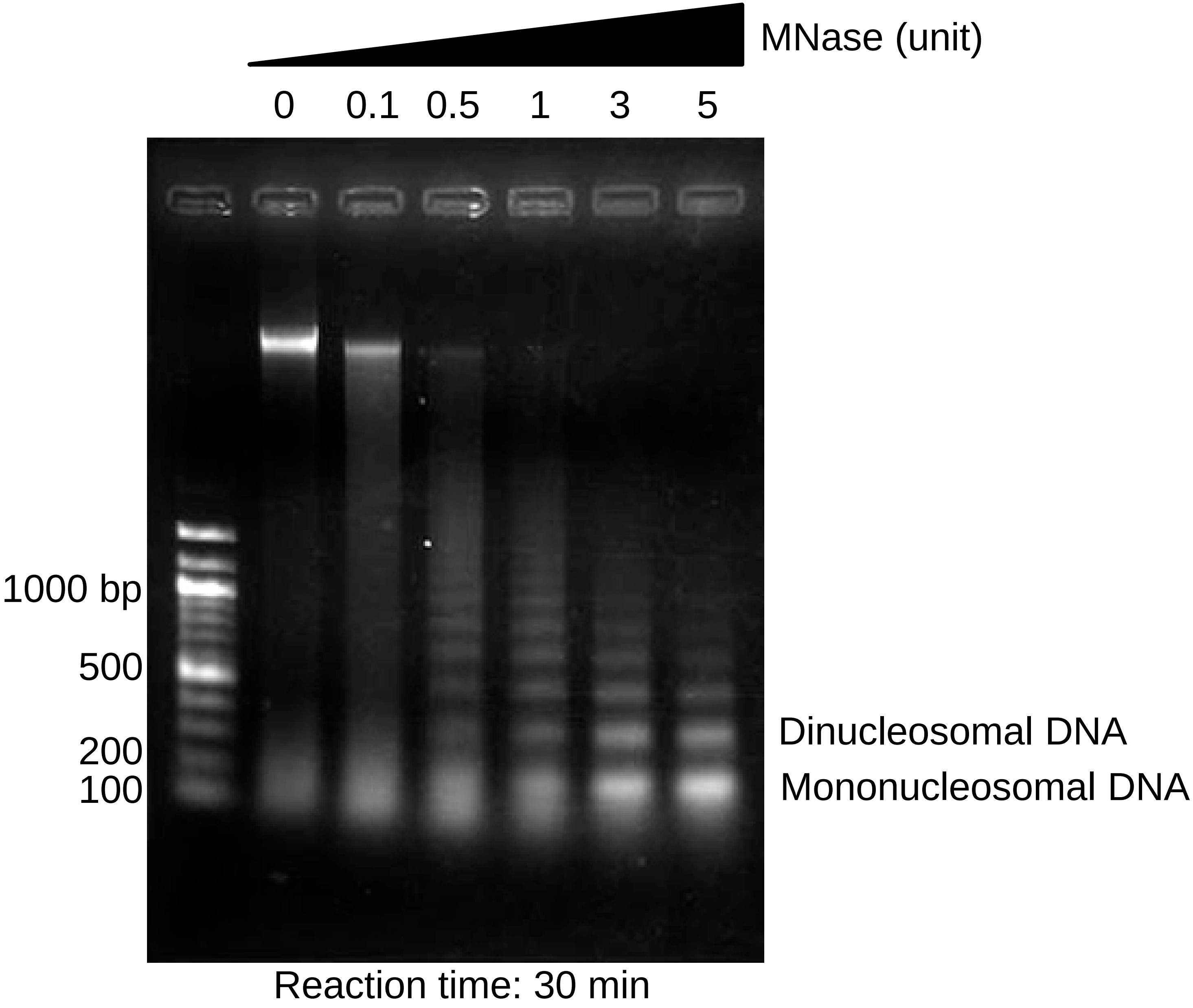

Supplementary Figure S2. Comparison of the mononucleosomal DNA lengths between fifty genes of highest and lowest levels of expression in the upstream and downstream regions of the transcription start sites.

The fifty highest and lowest genes were determined according to RPKM of each gene measured by mRNA-seq. We found no significant difference in the mononucleosomal DNA lengths between the two sets of genes showing clearly distinct levels of expression in either upstream or downstream regions of the transcription start sites. Mann-Whitney test yielded p-value > 0.7 and p-value > 0.11, respectively, for the upstream and downstream regions of the TSS.

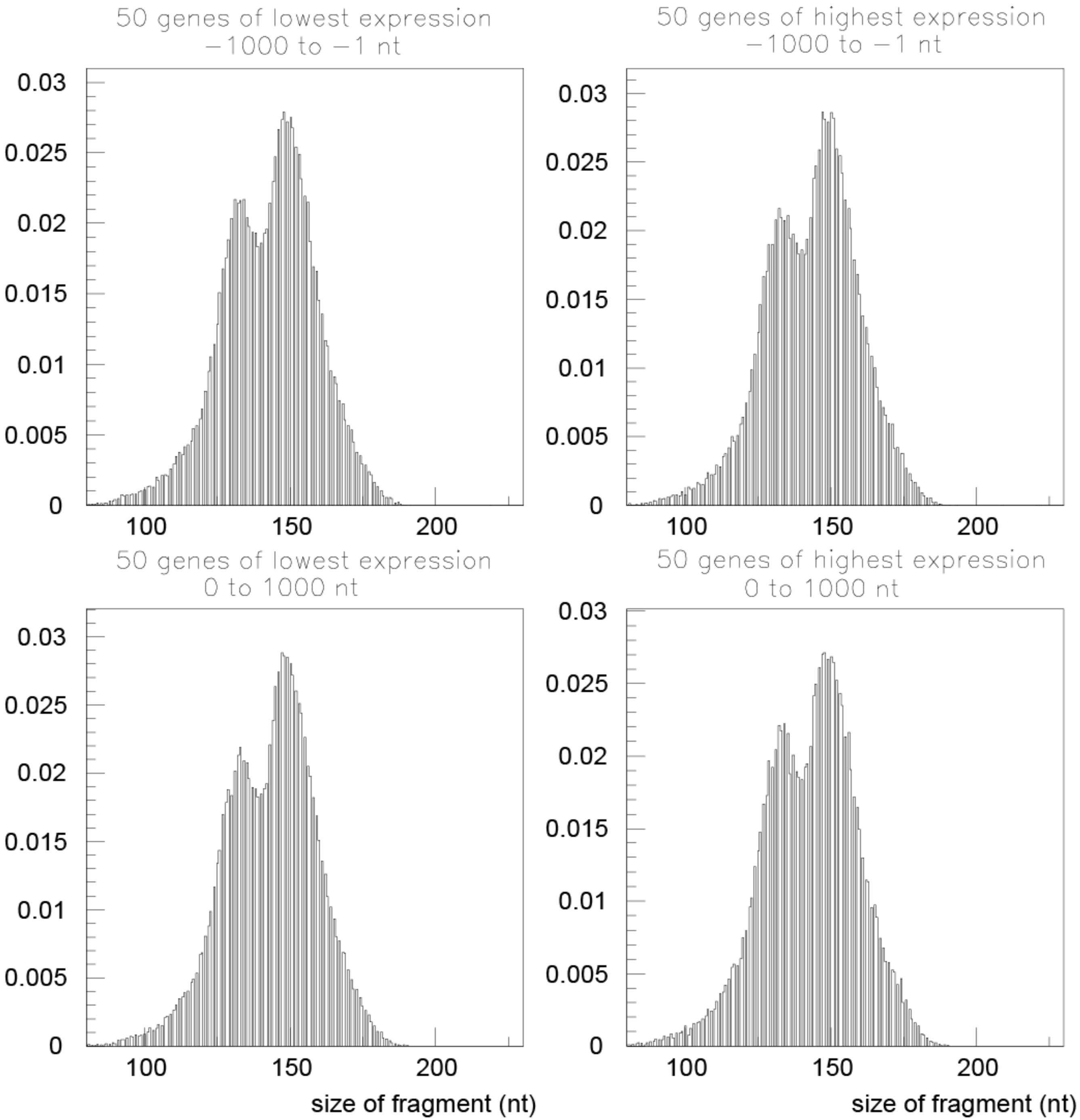

50 genes of lowest expression
−1000 to −1 nt
50 genes of highest expression
−1000 to −1 nt
50 genes of lowest expression
0 to 1000 nt
50 genes of highest expression
0 to 1000 nt
0.03
0.025
0.02
0.015
0.01
0.005
0
100
150
200
size of fragment (nt)

Supplementary Figure S3. Profiles of the midpoints of highly positioned mononucleosomes of *Mixia osmundae* around the translational termination sites.

The position profile of midpoints of highly positioned mononucleosomes in the vicinity of the 3’ end positions of complete CDSs is compared between the three groups of genes showing distinct levels of expression. The mononucleosomal midpoints of five or more piles were used.

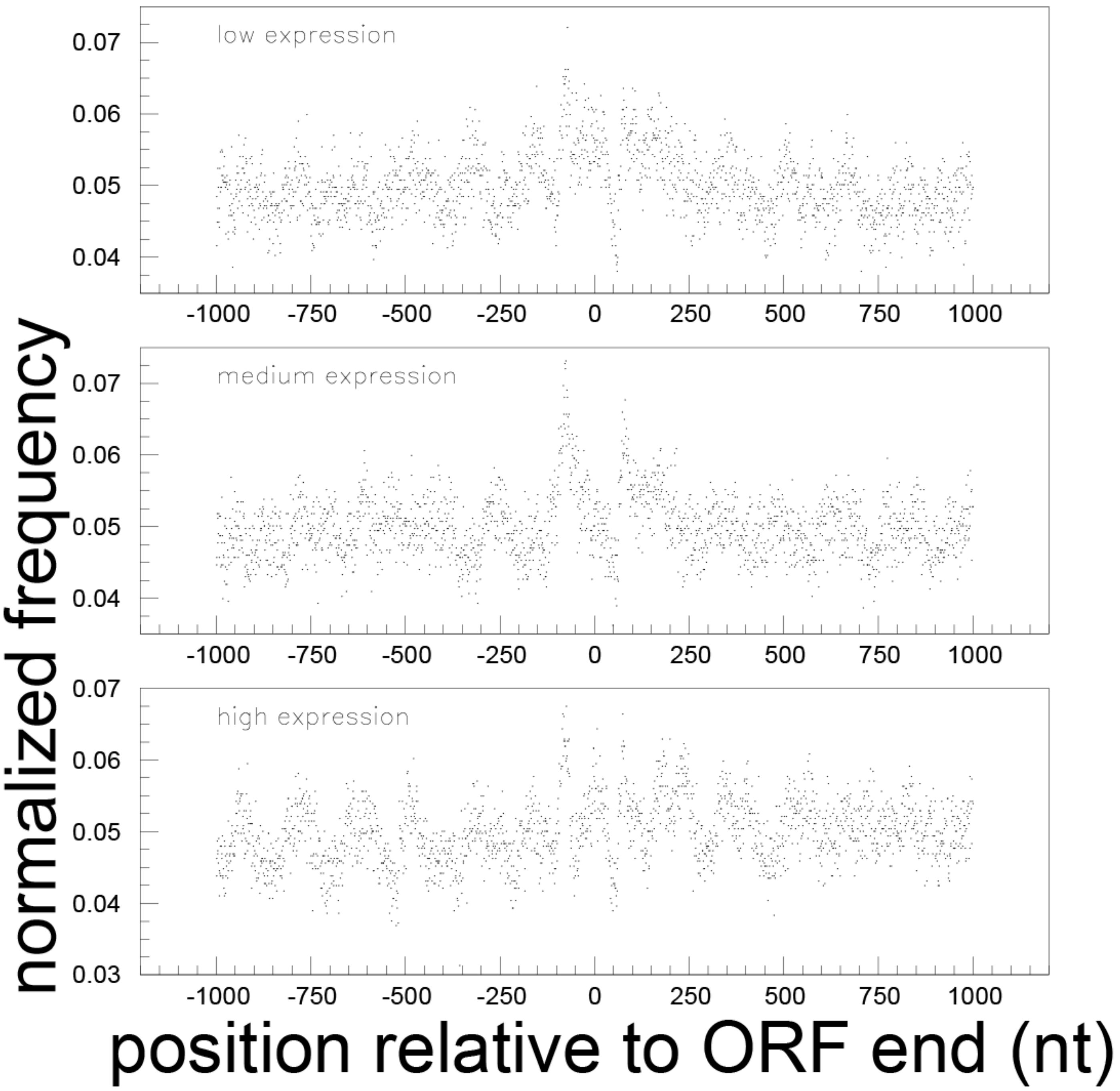

low expression
medium expression
high expression
0.07
0.06
0.05
0.04
0.03
-1000
-750
-500
-250
0
250
500
750
1000
normalized frequency
position relative to ORF end (nt)

Supplementary Figure S4. Profiles of the midpoints of highly positioned dinucleosomes of *Mixia osmundae* around the translational termination sites.

The position profile of midpoints of highly positioned dinucleosomes in the vicinity of the 3’ end positions of complete CDSs is compared between the three groups of genes showing distinct levels of expression. The dinucleosomal midpoints of five or more piles were used.

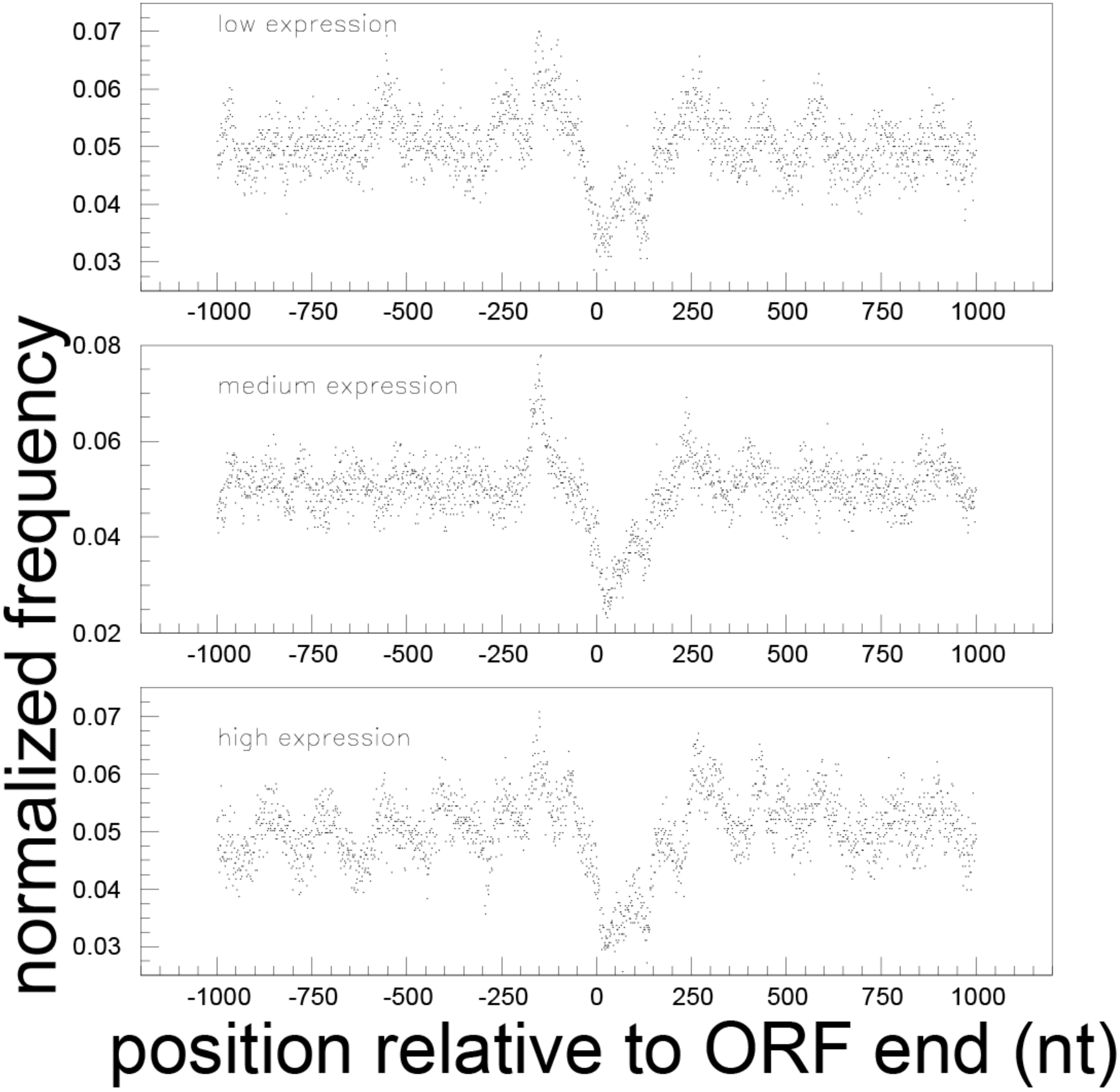

low expression
medium expression
high expression
0.07
0.06
0.05
0.04
0.03
0.08
0.02
-1000
-750
-500
-250
0
250
500
750
1000
normalized frequency
position relative to ORF end (nt)

Supplementary Figure S5. Illustration of the closest dinuleotide to the midpoint of a nucleosome mapped by a pair of Illumina GIIx reads.

The midpoint (dyad) of a nucleosomal DNA fragment mapped by a pair of Illumina GIIx reads is set to the position 0 in the coordinate. The dimer closest to the midpoint is the one located nearest to the position 0. For example, the closest AA dinucleotide to the midpoint is determined as follows. There are three AAs at positions -12, -4 and +10 in the above genomic regions flanking the midpoint. The AA closest to the midpoint is the one at position -4 in this case, and the distance between the dimer AA and the midpoint is 4 nt. If the dimer overlaps the midpoint or lies at positions -1 or 0, the distance is set to 0.

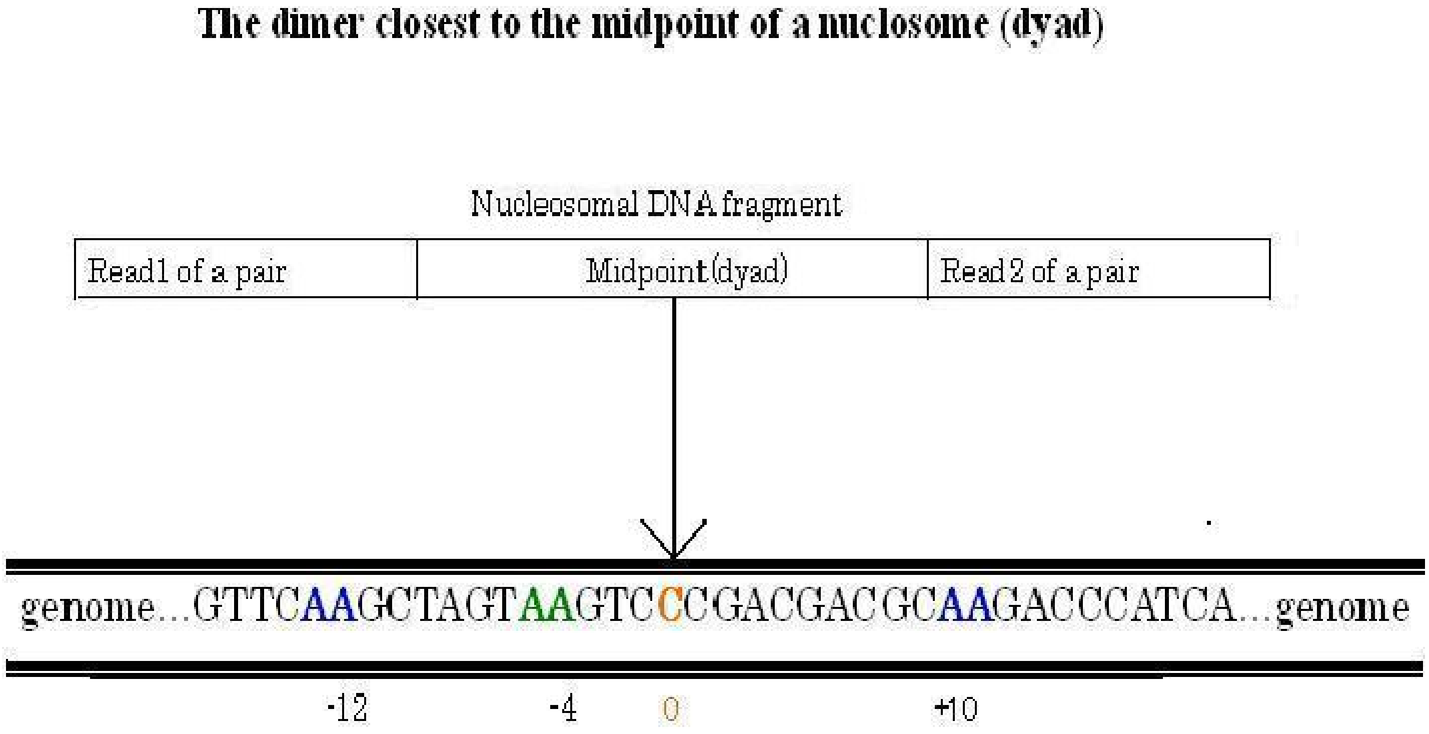

Supplementary Figure S6. Enriched and depleted dinucleotides within ±150 nt of the midpoints of highly positioned dinucleosomes of *Mixia osmundae.*

This is an extended version of Figure 8 which shows the profiles within ±80 nt from the midpoints of highly positioned dinucleosomes (5-pile subset). In this figure, the composite profiles are shown within ±150 nt of the midpoints.

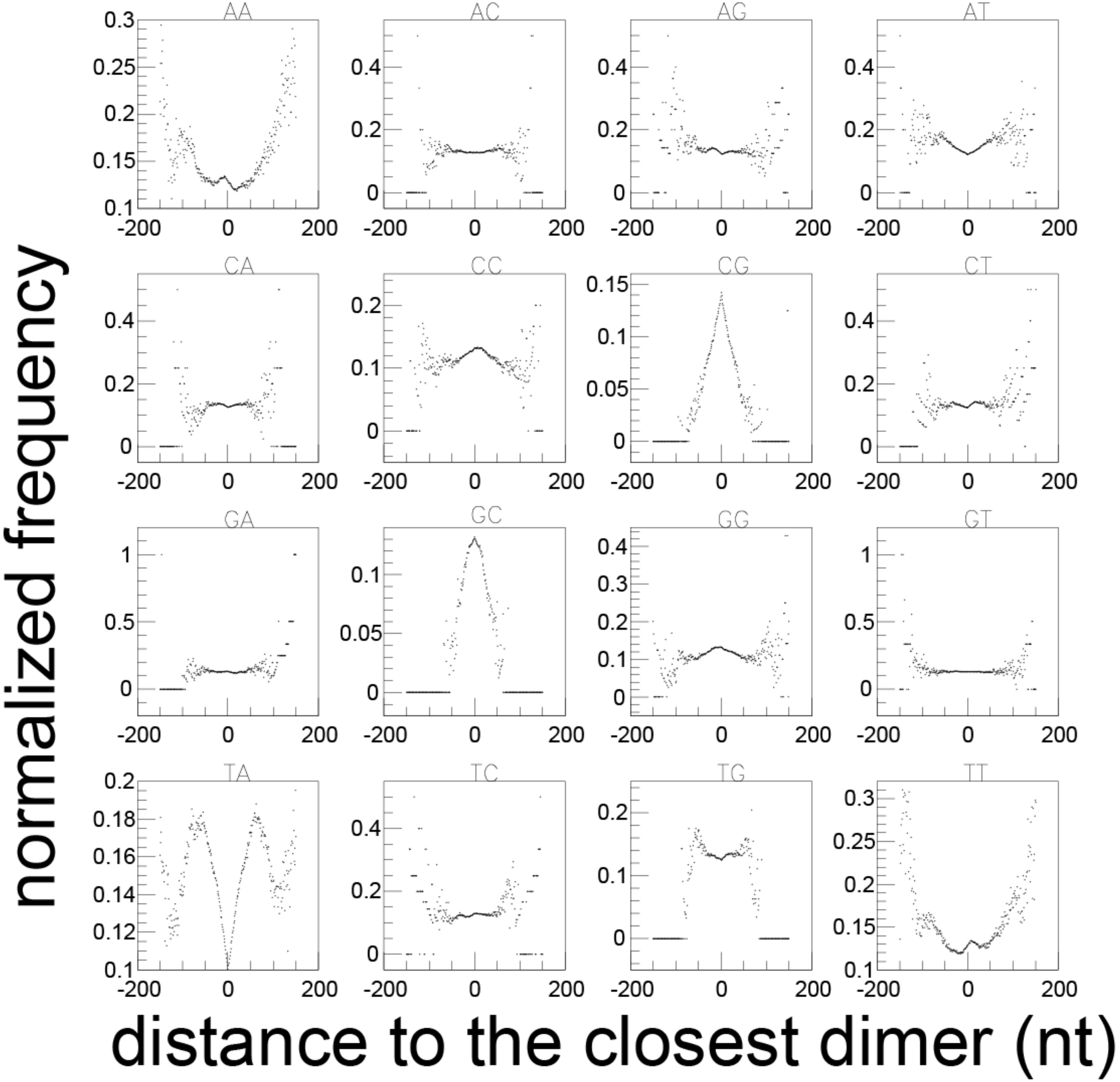

AA
AC
AG
AT
CA
CC
CG
CT
GA
GC
GG
GT
TA
TC
TG
TT
normalized frequency
distance to the closest dimer (nt)

Supplementary Figure S7. Dinucleotide frequencies in and around the midpoints of mononucleosomes.

The frequency of each of the 16 dinucleotides was computed in and around the midpoints of highly positioned mononucleosomes (pile-5 subset) and normalized by its genome wide frequency.

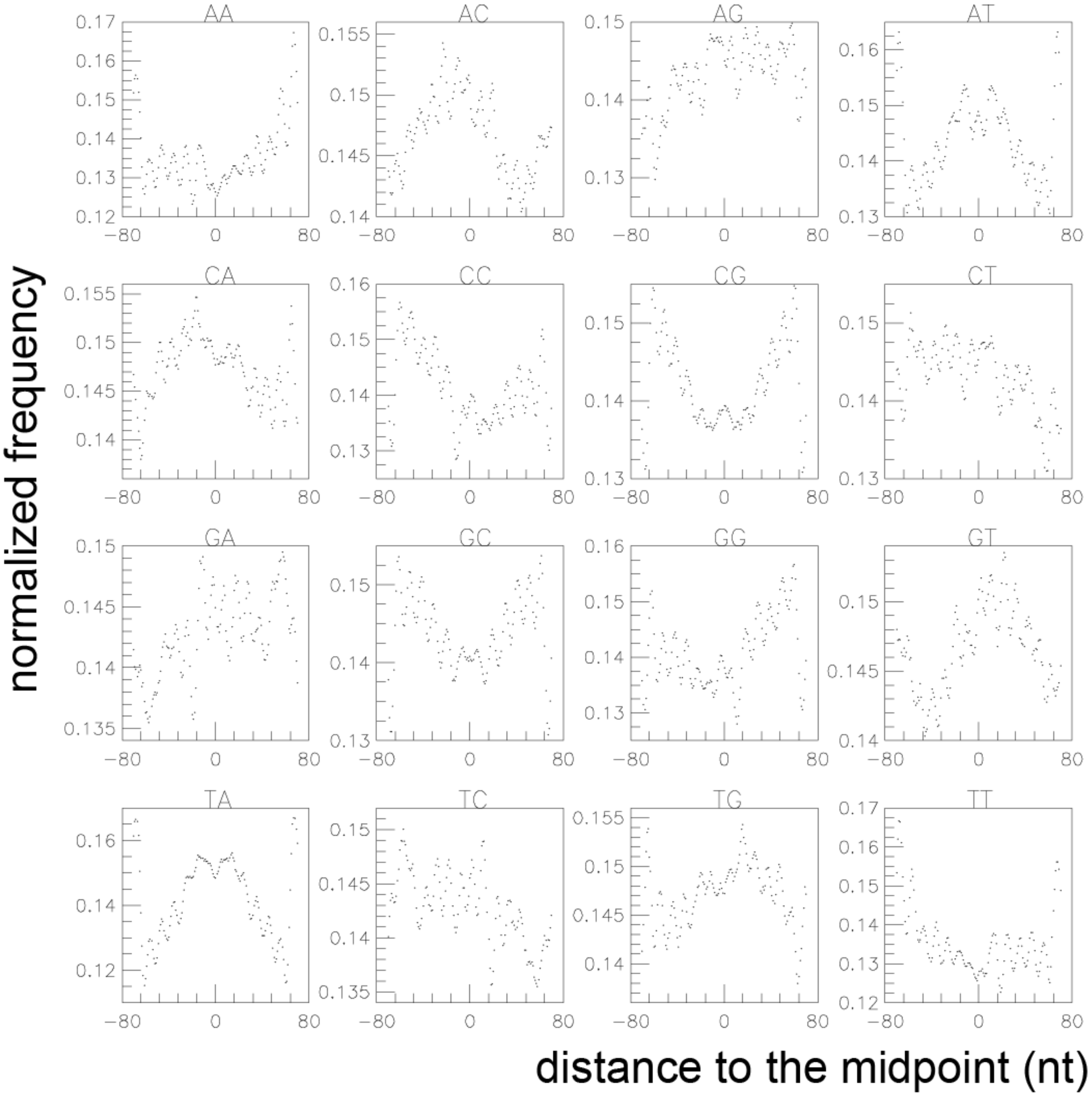

normalized frequency
distance to the midpoint (nt)
AA
AC
AG
AT
CA
CC
CG
CT
GA
GC
GG
GT
TA
TC
TG
TT

Supplementary Figure S8. Dinucleotide frequencies in and around the midpoints of dinucleosomes.

The frequency of each of the 16 dinucleotides was computed in and around the midpoints of highly positioned dinucleosomes (pile-5 subset) and normalized by its genome wide frequency.

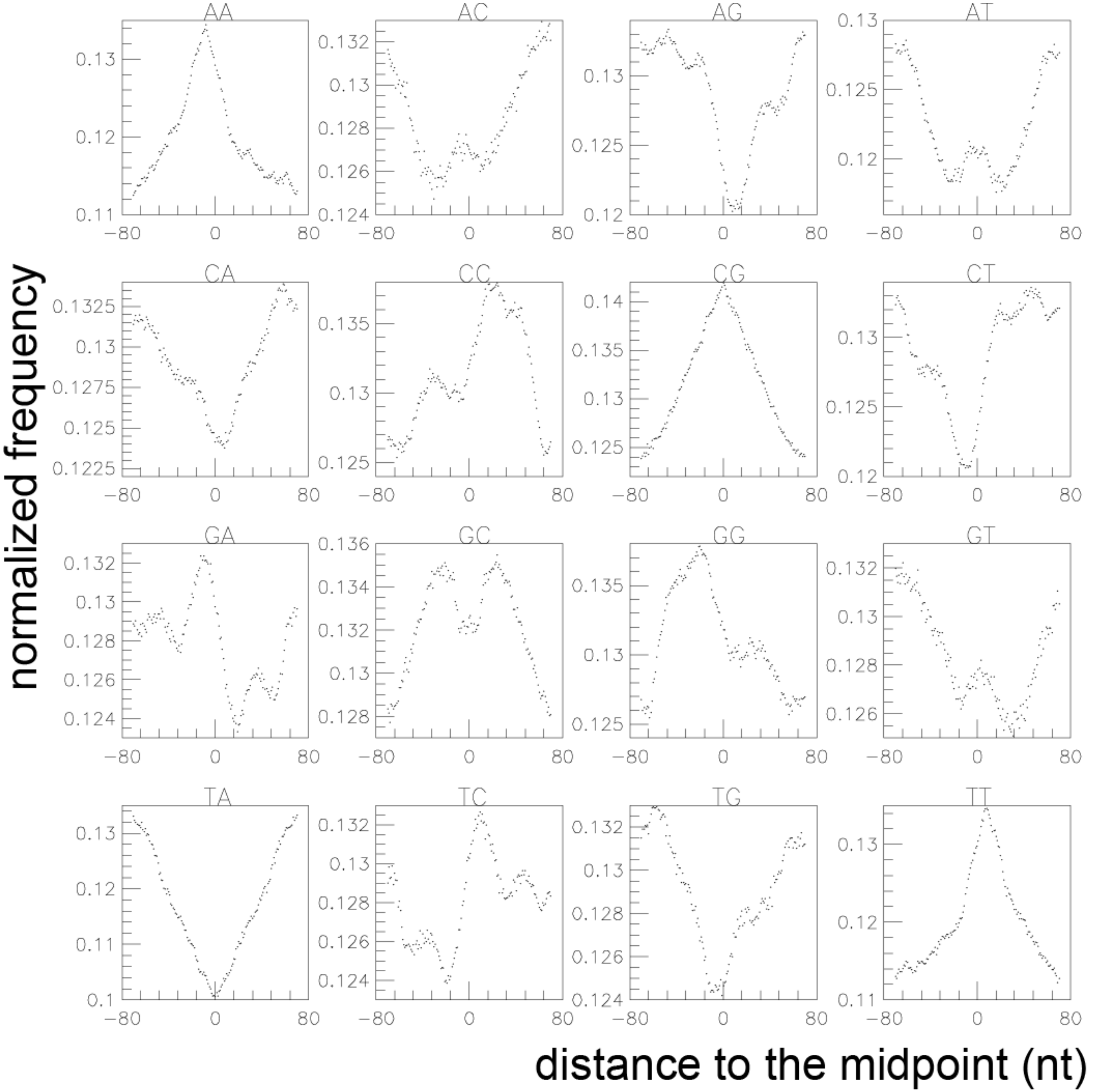
AA
AC
AG
AT
CA
CC
CG
CT
GA
GC
GG
GT
TA
TC
TG
TT
normalized frequency
distance to the midpoint (nt)

Supplementary Figure S9. Typical profiles of transcription start sites mapped around the genes predicted by AUGUSTUS.

Typical profiles of the TSS mapped near genes predicted in two distinct genome regions are shown together with profiles of midpoints of mono- and dinucleosomes. From the top, the midpoint profile of mononucleosomes (first lane), coverage profile of dinucleosomal DNA fragments (depth of mapped sequences) (second lane), midpoint profile of dinucleosomes (third lane), profile of the TSS mapped on the positive strand (fourth lane), genes predicted on the positive strand by AUGUSTUS (fifth lane), profile of the TSS mapped on the negative strand (sixth lane) and genes predicted on the negative strand by AUGUSTUS (bottom lane) are displayed.

Example 1

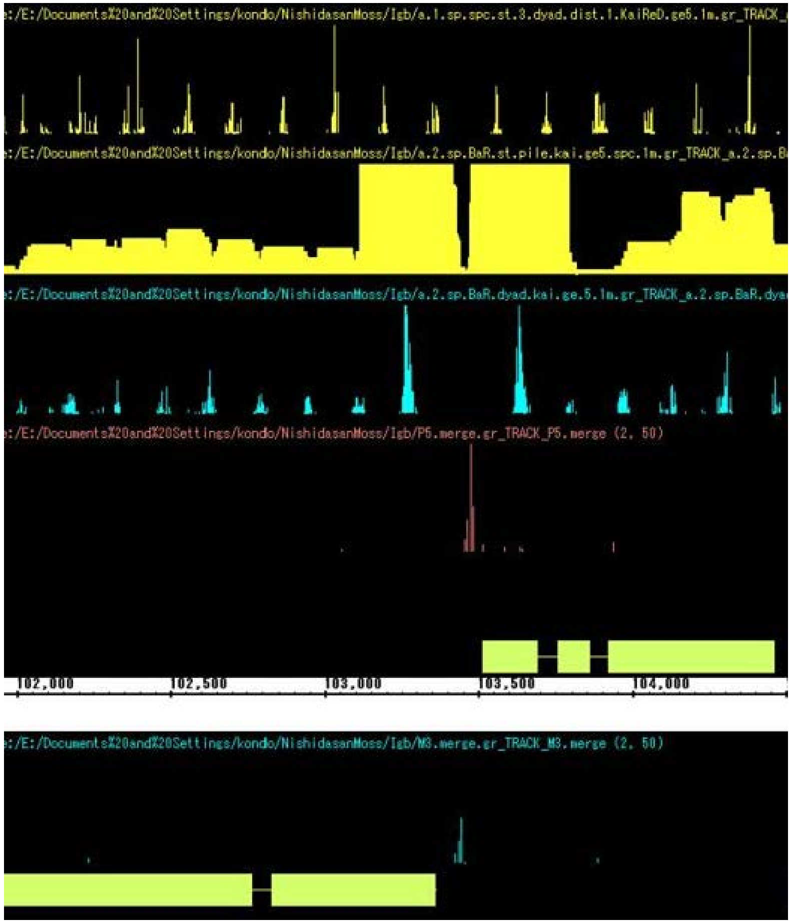

Example 2

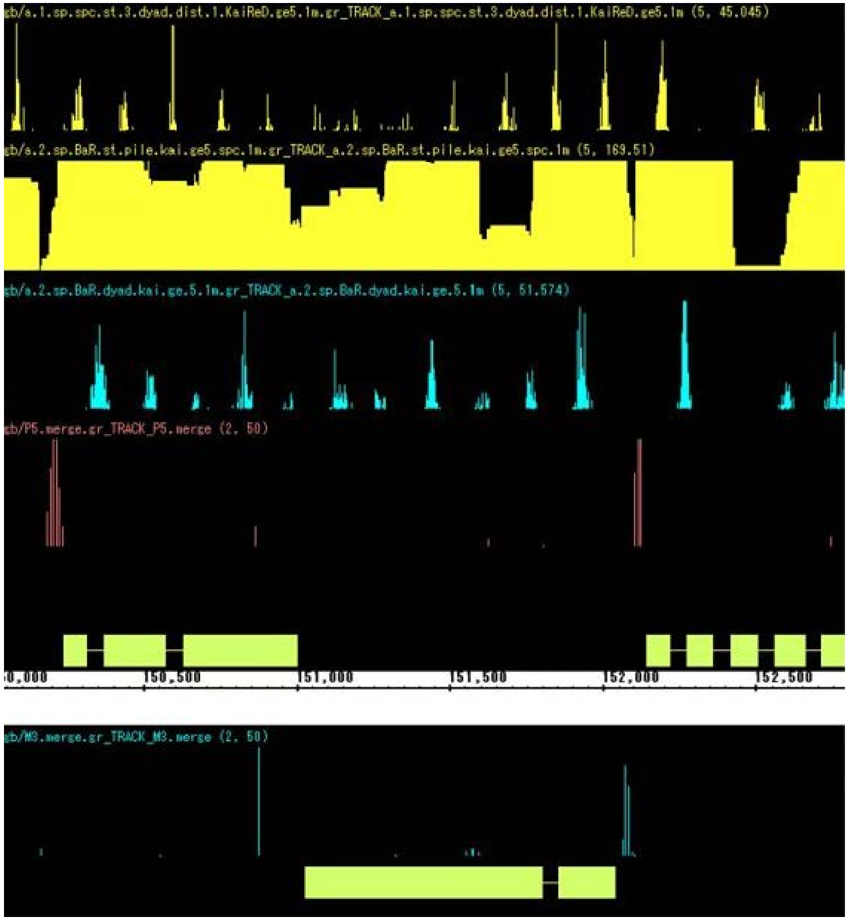

Supplement: Figures S1, S2, S3, S4, S5, S6, S7, S8, S9 [file rsob120043-s3.pdf]
